# Long-Lasting Protection of Activity of Nucleoside Reverse Transcriptase Inhibitors and Protease Inhibitors (PIs) by Boosted PI Containing Regimens

**DOI:** 10.1371/journal.pone.0050307

**Published:** 2012-11-26

**Authors:** Alexandra U. Scherrer, Jürg Böni, Sabine Yerly, Thomas Klimkait, Vincent Aubert, Hansjakob Furrer, Alexandra Calmy, Matthias Cavassini, Luigia Elzi, Pietro L. Vernazza, Enos Bernasconi, Bruno Ledergerber, Huldrych F. Günthard, the Swiss HIV Cohort Study (SHCS)

**Affiliations:** 1 Division of Infectious Diseases and Hospital Epidemiology, University Hospital Zürich, University of Zürich, Zürich, Switzerland; 2 Institute of Medical Virology, Swiss National Center for Retroviruses, University of Zürich, Zürich, Switzerland; 3 Laboratory of Virology, Geneva University Hospitals, Geneva, Switzerland; 4 Department Biomedicine, Haus Peterplatz, University of Basel, Basel, Switzerland; 5 Division of Immunology and Allergy, University Hospital Lausanne, Lausanne, Switzerland; 6 Division of Infectious Diseases, Clinic for Infectious Diseases, University Hospital Berne, University of Berne, Berne, Switzerland; 7 Division of Infectious Diseases, University Hospitals Geneva, Geneva, Switzerland; 8 Infectious Diseases Service, University Hospital Lausanne, Lausanne, Switzerland; 9 Division of Infectious Diseases and Hospital Epidemiology, University Hospital Basel, Basel, Switzerland; 10 Division of Infectious Diseases, Cantonal Hospital St. Gallen, St. Gallen, Switzerland; 11 Regional Hospital Lugano, Lugano, Switzerland; The University of North Carolina at Chapel Hill, United States of America

## Abstract

**Background:**

The accumulation of mutations after long-lasting exposure to a failing combination antiretroviral therapy (cART) is problematic and severely reduces the options for further successful treatments.

**Methods:**

We studied patients from the Swiss HIV Cohort Study who failed cART with nucleoside reverse transcriptase inhibitors (NRTIs) and either a ritonavir-boosted PI (PI/r) or a non-nucleoside reverse transcriptase inhibitor (NNRTI). The loss of genotypic activity <3, 3–6, >6 months after virological failure was analyzed with Stanford algorithm. Risk factors associated with early emergence of drug resistance mutations (<6 months after failure) were identified with multivariable logistic regression.

**Results:**

Ninety-nine genotypic resistance tests from PI/r-treated and 129 from NNRTI-treated patients were analyzed. The risk of losing the activity of ≥1 NRTIs was lower among PI/r- compared to NNRTI-treated individuals <3, 3–6, and >6 months after failure: 8.8% vs. 38.2% (p = 0.009), 7.1% vs. 46.9% (p<0.001) and 18.9% vs. 60.9% (p<0.001). The percentages of patients who have lost PI/r activity were 2.9%, 3.6% and 5.4% <3, 3–6, >6 months after failure compared to 41.2%, 49.0% and 63.0% of those who have lost NNRTI activity (all p<0.001). The risk to accumulate an early NRTI mutation was strongly associated with NNRTI-containing cART (adjusted odds ratio: 13.3 (95% CI: 4.1–42.8), p<0.001).

**Conclusions:**

The loss of activity of PIs and NRTIs was low among patients treated with PI/r, even after long-lasting exposure to a failing cART. Thus, more options remain for second-line therapy. This finding is potentially of high relevance, in particular for settings with poor or lacking virological monitoring.

## Introduction

The emergence of drug resistance is one of the major threats to successful antiretroviral therapy of infection with human immunodeficiency virus-1 (HIV-1) [1]. HIV-1 cannot be eradicated with today's antiretroviral treatment. The aim of therapy is thus to reduce morbidity and mortality by long-term inhibition of HIV-1 replication. Combination antiretroviral therapy (cART) is highly effective but viruses may start replicating if drug levels are too low (e.g. due to patients poor adherence or drug-drug interaction), concurrent infections or recent vaccinations. In these situations drug resistance mutations can accumulate [2]–[7]. To avoid long-lasting episodes of viral replication under cART and to detect a virological failure early, it is recommended to regularly monitor plasma viral load levels [8], [9]. However, in resource-limited settings the technical equipment, health care infrastructure and financial capacity are often lacking. Monitoring of cART is therefore often solely based on the measurements of CD4 cell counts. However, monitoring of treatment success by CD4 cell counts results in a significant delay to detecting treatment failure when compared to viral load monitoring and results in a higher burden of mutations [10], [11]. The accumulation of drug resistance-associated mutations reduces the options for subsequent successful second-line treatment dramatically. Therefore, it is important to identify cART combinations that result in long-lasting protection of the antiretroviral activity and to minimize the emergence of drug resistance mutations even if patients need to stay extended periods on a failing therapy [12].

We aimed to study the loss of genotypic activity at different time points after virological failure and the accumulation of mutations. We further sought to identify risk factors for early emergence of mutations and we aimed to describe antiretroviral treatments with a long-lasting protection of the genotypic activity after virological failure. To answer these questions, we used data from the Swiss HIV Cohort (SHCS) and the SHCS drug resistance database and compared sequences from genotypic drug resistance tests that were performed after patients had failed first-line cART.

## Methods

### Ethics statement

The SHCS has been approved by the following ethical committees of all participating institutions: Kantonale Ethikkommission Bern; Ethikkommission beider Basel; comité d'éthique du département de médicine de Hôpitaux Universitaires de Genève; commission d'éthique de la recherche clinique, Lausanne; comitato etico cantonale, Bellinzona; Ethikkommission des Kanton St.Gallens; and Ethik-Kommission Zürich, all Switzerland. Written informed consent has been obtained from all participants [13].

### Study population

We compared genotypic drug resistance tests from individuals included in the SHCS who failed first-line cART. The SHCS is a nationwide, multicenter, clinic-based cohort with continuous enrolment and semi-annual study visits. The last considered follow-up was the 18 October 2011. The SHCS drug resistance database is linked to the SHCS and includes >14,000 sequences from genotypic drug resistance tests performed by one of the four authorized laboratories in Switzerland [14]. Sequences are stored in SmartGene's (Zug, Switzerland) Integrated Database Network System (IDNS version 3.6.6).

### Patient selection and statistical analysis

We did a cross-sectional analysis and restricted our study to individuals who started first-line cART with nucleoside reverse transcriptase inhibitors (NRTIs) and either a ritonavir-boosted protease inhibitor (PI/r) or a non-nucleoside reverse transcriptase inhibitor (NNRTI) and who had a genotypic drug resistance test performed after virological failure but before treatment change to second-line cART. A treatment failure was defined if at least one HIV-1 RNA was detectable (≥50 copies/mL) after previous suppression (<50 copies/mL) or when individuals did not respond to the first-line cART for at least 180 days (no viral load <50 copies/mL). These individuals are further termed non-responders. If cART was changed before 90 days or if the last viral load during cART was undetectable, it was assumed that the treatment change was due to toxicity reasons and the next treatment was considered for analysis.

We calculated the time with replicating virus after virological failure until the resistance test was performed. The time with replicating virus was defined as the time period when patients had detectable viral loads (>50 copies/mL). If viral loads changed between two measurements from undetectable to detectable or vice versa, it was assumed that viruses were replicating half of the time. We grouped individuals with <3, 3–6 and >6 months with replicating virus.

Characteristics were compared with Fishers exact test (categorical variables) and Wilcoxon rank-sum test (continuous variables).

The loss of genotypic activity was estimated using the Stanford algorithm (version 6.1.1). The activity of a drug was defined as lost when the Stanford penalty score was ≥30 (http://hivdb.stanford.edu/). Drug resistance associated mutations were defined by IAS-USA [15]. Minor PI mutations were not considered for analysis. Thymidine-analogue mutations (TAMs) were categorized in TAM 1 (M41L, L210W, T215Y) and TAM 2 (D67N, K70R, T215F, K219E/Q).

We identified risk factors for an early accumulation of mutations (<6 months with replicating viruses). We performed univariable and multivariable logistic regression analyses. The following variables were included in the multivariable model: sex, transmission group, age, subtype, square root CD4 cell and viral load at the time when the resistance test was performed, NRTI treatment, PI/r or NNRTI use, and the year of cART initiation. Likelihood ratio tests did not indicate significant departures from linearity for continuous variables.

Adherence is an additional potential confounder. Self-reported adherence is documented only since May 2003 in the SHCS, therefore a sensitivity analysis including adherence data was performed with patients who failed cART after this date [16].

Not only the time with replicating virus but also the viral load might be predictive for the number of emerging mutations, therefore copy-years viremia was used in a sensitivity analysis instead of the time with replicating virus [17]. Copy-years viremia is a way to express the amount of exposure an individual has had to the virus over a period of time (akin to pack-years of smoking). The mean viral load of two successive measurements is multiplied by the time they are apart.

Statistical analyses were performed with Stata 11 SE software (StataCorp, College Station, TX, USA). All confidence intervals were two-sided, and the level of significance was set at 0.05.

## Results

### Baseline characteristics

We included 129 patients with a virological failure on a NNRTI-containing and 99 on a PI/r-containing cART (Table 1). The fraction of non-responders (patients who did not reach <50 copies/mL) was 20.2% and 22.2% (p = 0.745), respectively. Most baseline characteristics were similar between groups, although PI/r-treated patients started cART later (median: 2007 vs. 2004, p<0.001). The co-administered NRTIs varied slightly. The most commonly used NRTI combination in individuals treated with NNRTIs was zidovudine (AZT) and lamivudine (3TC) (41.1%). Most PI/r-treated patients received tenofovir (TDF) with either 3TC or emtricitabine (FTC) (45.5%, p = 0.061). The median time with replicating virus was 144.5 days (IQR: 87.5–233) and 141 days (IQR: 65–268) for NNRTI- and PI/r-treated patients (p = 0.573), respectively. Individuals were categorized by the time the resistance test was performed after virological failure: 34 and 34, 49 and 28, 46 and 37 treated with NNRTI or PI, respectively, had a resistance test performed after <3, 3–6 and >6 months with replicating virus. The median time with replicating virus in the category >6 months was similar between groups: 277.3 days (IQR: 226–506.5) and 292 days (IQR: 234.5–428) for NNRTI- and PI/r-treated patients (p = 0.916), respectively.

**Table 1:** Baseline characteristics of individuals who failed combination antiretroviral therapy (cART) containing a non-nucleoside reverse transcriptase inhibitor (NNRTI) or a ritonavir boosted protease inhibitor (PI/r).

	NNRTI				PI/r			
Characteristics	All	<3 months*	3–6 months*	>6 months*	All	<3 months*	3–6 months*	>6 months*
All patients	129 (100.0%)	34 (100.0%)	49 (100.0%)	46 (100.0%)	99 (100.0%)	34 (100.0%)	28 (100.0%)	37 (100.0%)
Sex								
Male	86 (66.7%)	22 (64.7%)	35 (71.4%)	29 (63.0%)	65 (65.7%)	21 (61.8%)	17 (60.7%)	27 (73.0%)
Female	43 (33.3%)	12 (35.3%)	14 (28.6%)	17 (37.0%)	34 (34.3%)	13 (38.2%)	11 (39.3%)	10 (27.0%)
Ethnicity								
White	83 (64.3%)	19 (55.9%)	34 (69.4%)	30 (65.2%)	64 (64.7%)	20 (58.8%)	17 (60.7%)	27 (73.0%)
Other	46 (35.7%)	15 (44.1%)	15 (30.6%)	16 (34.8%)	35 (35.4%)	14 (41.2%)	11 (39.3%)	10 (27.0%)
Transmission group								
homosexual men	38 (29.5%)	9 (26.5%)	19 (38.8%)	10 (21.7%)	38 (38.4%)	12 (35.3%)	7 (25.0%)	19 (51.4%)
Heterosexual	70 (54.3%)	23 (67.7%)	22 (44.9%)	25 (54.4%)	46 (46.5%)	18 (52.9%)	16 (57.1%)	12 (32.4%)
intravenous drug user	13 (10.1%)	1 (2.9%)	5 (10.2%)	7 (15.2%)	12 (12.1%)	4 (11.8%)	4 (14.3%)	4 (10.8%)
Other	8 (6.2%)	1 (2.9%)	3 (6.1%)	4 (8.7%)	3 (3.0%)	0 (0.0%)	1 (3.6%)	2 (5.4%)
Median age [IQR]	36 [31–46]	36.5 [29–52]	38 [31–45]	35 [32–42]	39 [33–45]	36 [31–41]	39 [32.5–45]	40 [36–48]
Median CD4 cell count (cells/µL) [IQR]	252 [114–450]	265.5 [139–446]	259 [110–405]	201 [101–464]	286 [117–432]	276.5 [123–407]	247 [96–454]	339 [127–471]
Median log_10_ HIV-1 RNA (copies/mL) [IQR]	5.0 [4.4–5.4]	4.9 [4.4–5.3]	5.1 [4.6–5.7]	5.0 [4.4–5.3]	5.0 [4.6–5.7]	5.0 [4.6–5.7]	5.2 [4.6–5.8]	5.0 [4.2–5.4]
Subtype								
B	81 (62.8%)	20 (58.8%)	32 (65.3%)	29 (63.0%)	63 (63.6%)	20 (58.8%)	14 (50.0%)	29 (78.4%)
non-B	48 (37.2%)	14 (41.2%)	17 (34.7%)	17 (37.0%)	36 (36.4%)	14 (41.2%)	14 (50.0%)	8 (21.6%)
Median Year of cART initiation [IQR]	2004 [2002–2007]	2006 [2003–2008]	2005 [2002–2007]	2003 [2001–2005]	2007 [2004–2008]	2007 [2003–2009]	2007 [2005–2009]	2006 [2003–200]]
NRTI treatment								
3TC TDF	18 (13.9%)	3 (8.8%)	7 (14.3%)	8 (17.4%)	5 (5.0%)	2 (5.9%)	2 (7.1%)	1 (2.7%)
FTC TDF	22 (17.1%)	9 (26.5%)	11 (22.4%)	2 (4.3%)	40 (40.4%)	9 (26.5%)	13 (46.4%)	18 (48.6%)
ABC 3TC	13 (10.1%)	8 (23.5%)	3 (6.1%)	2 (4.3%)	13 (13.1%)	6 (17.6%)	4 (14.3%)	3 (8.1%)
AZT 3TC	53 (41.1%)	10 (29.4%)	18 (36.7%)	25 (54.4%)	26 (26.3%)	13 (38.2%)	5 (17.9%)	8 (21.6%)
Other	23 (17.8%)	4 (11.8%)	10 (20.4%)	9 (19.6%)	15 (15.2%)	4 (11.8%)	4 (14.3%)	7 (18.9%)
NNRTI treatment								
Efavirenz	96 (74.4%)	24 (70.6%)	35 (71.4%)	37 (80.4%)				
Nevirapine	33 (25.6%)	10 (29.4%)	14 (28.6%)	9 (19.6%)				
PI/r treatment								
Lopinavir/r					50 (50.5%)	19 (55.9%)	14 (50.0%)	17 (46.0%)
Atazanavir/r					30 (30.3%)	9 (26.5%)	7 (25.0%)	14 (37.8%)
Other					19 (19.2%)	6 (17.6%)	7 (25.0%)	6 (16.2%)
Type of failure								
Failure after suppression	103 (79.8%)	32 (94.1%)	36 (73.5%)	35 (76.1%)	77 (77.8%)	31 (91.2%)	22 (78.6%)	24 (64.9%)
Non-responder	26 (20.2%)	2 (5.9%)	13 (26.5%)	11 (23.9%)	22 (22.2%)	3 (8.8%)	6 (21.4%)	13 (35.1%)

* Time with replicating virus after treatment failure. Abbreviations: IQR, interquartile range; NRTI, nucleoside reverse transcriptase inhibitor; 3TC, lamivudine; FTC, emtricitabine; TDF, tenofovir; ABC, abacavir; AZT, zidovudine.

The median viral load at the time when the genotypic resistance test was performed was considerably higher among patients treated with a NNRTI compared to a PI/r (HIV-1 RNA: log_10_ 3.5 copies/mL [IQR: 2.8–4.6] vs. log_10_ 2.8 copies/mL [IQR: 2.3–3.6], p<0.001). In addition, the copy-years viremia was also substantially higher in patients treated with NNRTI (641.2 years * copies/mL [IQR: 114.5–8348.1], p<0.001) compared to PI/r (216.9 years * copies/mL [IQR: 33.9–1454.6]).

The numbers of transmitted NRTI mutations were similar in NNRTI- and PI/r treated individuals. A resistance test had been performed prior to any cART initiation among 146 of 228 individuals. The prevalence of transmitted NRTI mutations was 1.5% and 5.0% in PI/r and NNRTI-treated individuals (p = 0.378), respectively. Restricting the analysis to patients with known baseline resistance data and without transmitted drug resistance mutations did not alter conclusions (data not shown).

Adherence data was available for 157 of 169 (92.9%) individuals who failed cART after May 2003. Adherence was similar between patients treated with PI/r- or NNRTI-containing cART: 76.9% and 79.8% (p = 0.503) never missed a drug dose six months before reporting adherence.

### Loss of genotypic activity of NRTIs

**Figure 1:**
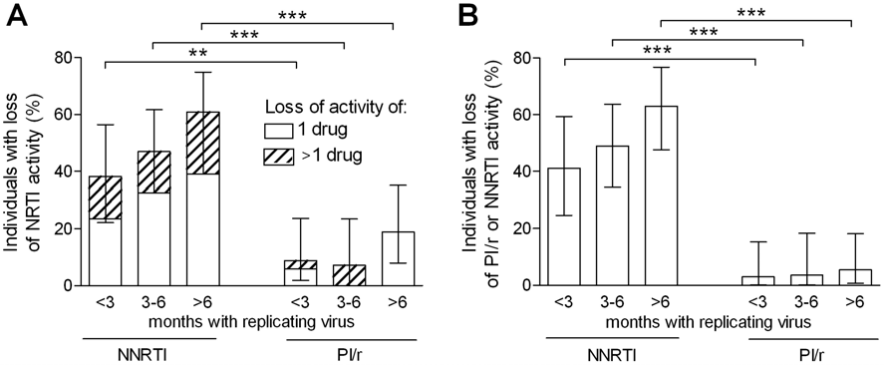
Loss of genotypic activity. Loss of genotypic activity among patients treated with non-nucleoside reverse transcriptase inhibitors (NNRTIs) or ritonavir-boosted protease inhibitors (PI/r). A) Loss of activity of 1 NRTI (open bar section) or >1 NRTIs (hatched bar section). B) Loss of activity of a NNRTI or PI/r. 95% confidence intervals are indicated. *** Fisher exact p<0.001, ** p<0.01.

The loss of genotypic activity of NRTIs was considerably higher in patients treated with NNRTIs compared to PI/r. The loss of genotypic activity of NRTIs was already very high <3 months after failure when patients had been treated with NNRTIs (38.2%) whereas PI/r-treated patients rarely accumulated NRTI mutations in this time period (8.8%, p = 0.009). The loss of genotypic activity of NRTIs remained considerably higher among patients treated with NNRTIs compared to PI/r also after long-lasting exposure to the failing regimen: 46.9% vs. 7.1% (p<0.001) between 3–6 months and 60.9% vs. 18.9% (p<0.001) after >6 months with replicating virus. The loss of activity of more than one NRTI was quite rare in the PI/r group for all time points: 2.9%, 7.1%, 0% when compared to the NNRTI group: 14.7% (p = 0.197), 14.3% (p = 0.474), 21.7% (p = 0.002) at <3, 3–6 and >6 months after failure (Figure 1 A).

We excluded non-responders (n = 48) from a sensitivity analysis. The results remained more favorable for the PI/r group: 37.5% vs. 9.7% (p = 0.016), 38.9% vs. 9.1% (p = 0.016), 57.1% vs. 16.7% (p = 0.003) NNRTI- and PI-treated individuals lost the activity of ≥1 NRTI after <3, 3–6, >6 months with replicating virus. Results were confirmed if the analysis was restricted to the 113 patients who truly failed first-line cART (patients who changed a successful cART regimen due to toxicity reasons were excluded) although the sample size was reduced: 23.1% vs. 38.5% (p = 0.673), 8.3% vs. 48.0% (p = 0.027), 22.2% vs. 65.6% (p = 0.007) after <3, 3–6, >6 months with replicating virus lost the activity of ≥1 NRTI in the PI/r and NNRTI group, respectively. The treatment with more potent NRTI combinations could be a potential explanation for the lower accumulation of NRTI mutations. PI/r-treated individuals received more often the modern and well tolerated NRTI combination TDF/FTC (40.4%) compared to NNRTI-treated individuals (17.1%). In a sensitivity analysis, we only included patients who were treated with the TDF/FTC NRTI combination (n = 62). Also in this sub-group, the loss of activity of ≥1 NRTI was higher in NNRTI compared to PI/r-treated individuals, 26.1% compared to 5% (p = 0.043), respectively.

Higher copy-years viremia among patients treated with NNRTI would be a potential explanation for faster accumulation of mutations. We repeated the analysis and classified patients in 3 groups based on the copy-years viremia instead of the time with replicating virus. Each group contained 76 patients with copy-years viremia <115, 115–1491,>1491 years * copies/mL. The loss of genotypic activity of ≥1 NRTIs was 60.6% vs. 7.0% (p<0.001), 51.2% vs. 15.2% (p = 0.002) and 41.5% vs. 17.4% (p = 0.064) in group 1 to 3 among NNRTI and PI/r treated individuals, respectively. These findings demonstrate that the higher loss of activity in NNRTI-treated patients cannot solely be explained by a more intense viral replication.

### Loss of genotypic activity of PIs and NNRTIs

The loss of genotypic activity of NNRTIs occurred very early after treatment failure whereas the emergence of PI/r resistance was very rare even after long-lasting exposure to failing cART. The percentage of patients who have lost PI/r activity was 2.9%, 3.6%, 5.4% <3, 3–6 and >6 months compared to 41.2%, 49.0%, 63.0% of those who have lost NNRTI activity (all p<0.001) (Figure 1 B). Results were similar when individuals were categorized according to copy-years viremia strata: 7.0% vs. 57.6%, 0% vs. 44.2%, 4.4% vs. 54.7% (all p<0.001).

Excluding non-responders or studying exclusively patients who failed first-line treatment confirmed these results (data not shown).

### Emerging mutations

**Figure 2:**
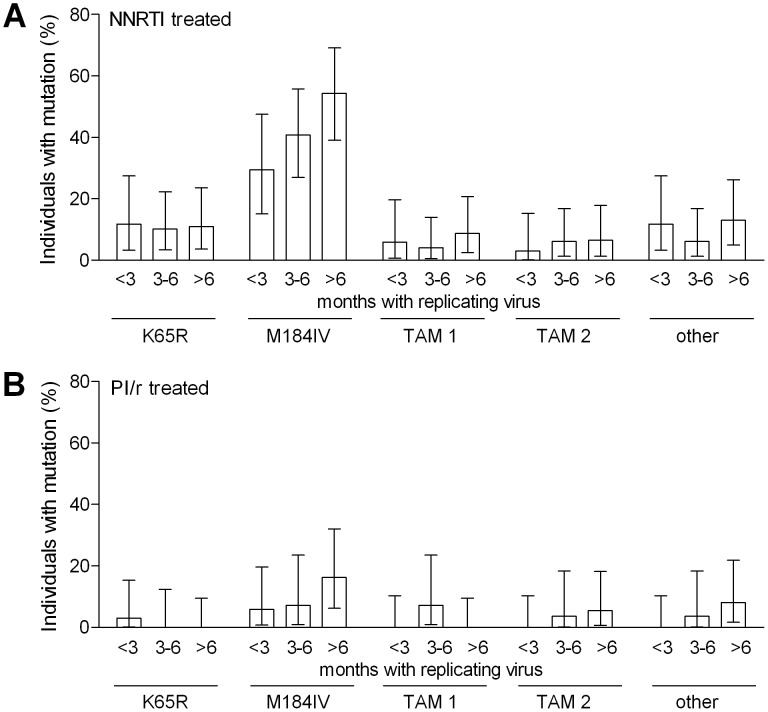
Emergence of mutations. Accumulation of nucleoside reverse transcriptase inhibitor (NRTI) mutations after virological failure on combination antiretroviral therapy containing A) non-nucleoside reverse transcriptase inhibitors (NNRTIs) or B) ritonavir-boosted protease inhibitors (PI/r): K65R, M184I/V, thymidine analogue mutations (TAM) 1 or 2, and other NRTI mutations defined by IAS-USA [15]. 95% confidence intervals are indicated.

The most common cause for the loss of genotypic activity of NRTIs was the emergence of M184V. It occurred in 36.4% of NNRTI- and in only 9.1% of PI/r-treated individuals (p<0.001). The prevalence of additional NRTI mutations was also much higher in the NNRTI when compared to the PI/r group: K65R 10.9% vs. 1.0% (p = 0.003), M184I 7.0% vs. 1.0% (p = 0.029), T215Y 5.4% vs. 1.0% (p = 0.072). All other mutations had a prevalence of <5% in both groups (Figure 2).

The loss of NNRTI activity was mainly caused by the emergence of K103N (27.9%), Y181C (10.9%) or G190A (6.2%). The most common PI mutations in PI/r-treated individuals were L90M (3.0%), I84V (2.0%) and M46I (2.0%).

### Risk factors for the early emergence of NRTI mutations

Exposure to NNRTIs was the major risk factor for an early accumulation of NRTI mutations. The odds ratio (OR) was 10.6 (95% CI: 3.9–29.1) and 13.3 (95% CI: 4.1–42.8) in univariable and multivariable models, respectively. High CD4 cell count at the time of the resistance test was the only other factor that had a weak association with a later accumulation of NRTI mutations (univariable OR: 0.9 (95% CI: 0.9–1.0), multivariable OR: 0.9 (95% CI: 0.8–1.0). No other factor was significantly associated in the multivariable model (Table 2).

**Table 2:** Univariable and multivariable logistic regression analyzing risk factors for an early (<6 months with replicating viruses) emergence of ≥1 nucleoside reverse transcriptase (NRTI) mutations (n = 145).

Characteristics	Individuals with ≥1 NRTI mutation/failures (%)	Univariable odds ratio (95% CI)	P	Multivariable odds ratio (95% CI)	P
Sex					
male	30/95 (31.6%)	1 (Ref)		1 (Ref)	
female	15/50 (30.0%)	0.9 (0.4–2.0)	0.845	1.3 (0.5–3.5)	0.663
Transmission group					
non-IDU	39/131 (29.8%)	1 (Ref)		1 (Ref)	
IDU	6/14 (42.9%)	1.8 (0.6–5.4)	0.319	2.4 (0.5–12.0)	0.274
Age		1.0 (0.9–1.0)	0.343	1.0 (0.9–1.0)	0.175
Subtype					
non-B	15/59 (25.4%)	1 (Ref)		1 (Ref)	
B	30/86 (34.9%)	1.6 (0.8–3.3)	0.228	1.6 (0.6–4.4)	0.401
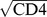 cells/µL		0.9 (0.9–1.0)	0.014	0.9 (0.8–1.0)	0.024
log_10_ HIV RNA		1.5 (1.1–2.1)	0.013	1.0 (0.6–1.5)	0.907
NRTI backbone					
3TC TDF	8/14(57.1%)	1 (Ref)		1 (Ref)	
FTC TDF	8/42(19.1%)	0.2 (0.0–0.7)	0.009	0.3 (0.1–1.8)	0.195
ABC 3TC	4/21(19.1%)	0.2 (0.0–0.8)	0.025	0.2 (0.0–1.3)	0.093
AZT 3TC	15/46(32.6%)	0.4 (0.1–1.2)	0.105	0.4 (0.1–1.9)	0.222
other	10/22(45.5%)	0.6 (0.2–2.4)	0.495	0.5 (0.1–2.9)	0.460
Second drug class					
PI/r	5/62 (8.1%)	1 (Ref)		1 (Ref)	
NNRTI	40/83 (48.2%)	10.6 (3.9–29.1)	<0.001	14.3 (4.3–47.5)	<0.001
Year of cART start		0.9 (0.8–1.0)	0.010	1.0 (0.8–1.2)	0.698

Abbreviation: CI, confidence interval; IDU, intravenous drug user; FTC, emtricitabine; 3TC, lamivudine; ABC, abacavir; AZT, zidovudine; TDF, tenofovir; PI/r, ritonavir-boosted protease inhibitor; NNRTI, non-nucleoside reverse transcriptase inhibitor.

## Discussion

We showed that cART containing PI/r results in a long-lasting protection of the activity of NRTIs and PI/r during sustained viral replication under therapy. In contrast, if patients are treated with NNRTI-based cART, NRTI mutations emerge much earlier and in larger numbers. These findings are of importance both, for resource-rich and resource-limited settings. In resource-rich settings, treatment failures are usually diagnosed quite early because of frequent viral load monitoring. In resource-limited settings patients often stay a long time on a failing regimen due to lacking or only infrequent viral load monitoring. In both situations, more options remain for second-line treatment if patients receive a PI/r-based cART as first-line therapy.

Previous randomized and observational studies showed that the failure rate between PI/r and NNRTI is comparable in most cases but fewer mutations emerge when patients fail a PI/r treatment [14], [18]–[21]. Mainly the activity of PI/r is well protected but also the activity of NRTIs [21]–[25]. In extension to these earlier data, we demonstrated in our study that this effect is long-lasting. After more than 6 months sustained viral replication on PI/r-containing cART, the loss of activity of ≥1 NRTI is only 18.9% compared to 60.9% on NNRTI-containing cART. This finding is of particular interest for resource-limited settings without virological monitoring where high numbers of NRTI mutations, mainly M184V, and NNRTI mutations are common in first-line failures treated with NNRTI-containing cART [26]–[28]. The number of accumulating mutations can be reduced when virological monitoring is performed [10], [29]. However, in many settings infrastructure and costs do not allow virological monitoring at regular intervals [12], therefore the use of PI/r as first-line therapy might be an interesting alternative in order to save more options for second-line treatment. Although drug resistance is an important factor to be considered, co-formulations, simplicity of administration, costs, drug-drug interactions, toxicity and adverse events need also to be taken into account for the choice of first-line treatment [8].

In general, it is astonishing how few mutations were observed overall in the 228 patients of the study who have failed therapy. Only 43% of patients had any drug resistance-associated mutation detected [21]. Missing drug pressure due to poor adherence could be a possible explanation for the low prevalence of mutations but it is probably not the major reason because >75% of patients reported to have an excellent adherence. Nevertheless, the prevalence of resistance might be underestimated. Currently used genotypic resistance tests have a population detection limit of only ∼20%. Additional resistant virus variants might be present at lower levels [30]–[33]. The late and rare occurrence of PI/r mutations can be explained by their high genetic barrier compared to NNRTIs [34]. However, the mechanism explaining the lack of resistance to co-administered NRTIs remains unknown. It can be speculated that the two drug classes may have different activities in different anatomical compartments [35], with regards to free versus cell-cell virus transmission [36] so that the activity of PI/r might be sufficient to suppress NRTI resistant strains to undetectable levels [21]. It could also be possible that NNRTIs, as they target the same gene as NRTIs, might select for yet unidentified compensatory mutations in the RT connection-, respectively, RNase H-domain of the pol gene [37], [38], subsequently leading to more rapid emergence of NRTI mutations [39], [40]. In theory, the presence of minority variants harboring NNRTI- or NRTI-drug resistant mutations, which have been detected in drug naive HIV-1 infected patients, could have a more severe impact in a regimen that contains a “low genetic barrier” drug rather than a PI/r. This aspect cannot be excluded in the present study. [32], [41]. Poorer adherence in the PI/r-treated group could also possibly explain the differences (no selective drug pressure from NRTIs) but adherence was excluded as potential bias in a sensitivity analysis. In addition, different NRTI backbones in PI/r- and NNRTI-treated individuals might have influenced our results [42], [43]. To disprove this concern, we performed a sensitivity analysis only including patients with a TDF/FTC backbone and we adjusted the logistic regression for the NRTI backbone.

Although our study initially considered 5959 patients who started first-line cART, only 228 individuals qualified for our study. The sample size was too small to compare different treatment regimens in more detail. Unfortunately, sufficient longitudinal resistance data from our patients were not available; otherwise dynamics of evolution of individual drug resistance mutations could have been investigated in more detail. In addition, we cannot exclude that there are resistance associated mutations outside the sequenced region. No phenotypic resistance tests were available that could prove that viruses which do not harbor any mutations are really sensitive to the drugs.

In conclusion, PI/r containing cART leads to long-lasting protection of the activity of NRTIs and PI/r despite ongoing viral replication after virological failure. Accumulation of drug resistance mutations against all three drugs of the regimen is slower and less frequent when compared to NNRTI-containing regimens, thus retaining more options for second-line therapy. These findings are of high relevance for settings, which lack the opportunities for regular virological monitoring and where the use of PI/r as first-line therapies should be considered.
