# Correction: Long-Lasting Protection of Activity of Nucleoside Reverse Transcriptase Inhibitors and Protease Inhibitors (PIs) by Boosted PI Containing Regimens

**DOI:** 10.1371/annotation/78eea40b-1982-4734-b32e-f94ec6d7b85a

**Published:** 2013-07-25

**Authors:** Alexandra U. Scherrer, Jürg Böni, Sabine Yerly, Thomas Klimkait, Vincent Aubert, Hansjakob Furrer, Alexandra Calmy, Matthias Cavassini, Luigia Elzi, Pietro L. Vernazza, Enos Bernasconi, Bruno Ledergerber, Huldrych F. Günthard, the Swiss HIV Cohort Study (SHCS)

The following text is missing from the Competing Interest Statement:

The grant from the Union Bank of Switzerland is unrestricted and not related to any employment, consultancy, patents, products in development or marketed products etc. This does not alter our adherence to all the PLoS ONE policies on sharing data and materials. 

